# Endoscopic treatment of obstructive ureterohydronephrosis in children

**DOI:** 10.11604/pamj.2020.36.260.24828

**Published:** 2020-08-10

**Authors:** Charikleia Demiri, Vassilis Lambropoulos, Vasileios Mouravas, Chrysostomos Kepertis, Dimitrios Godosis, Maria Tsopozidi, Ioannis Spyridakis

**Affiliations:** 1 Second Department of Paediatric Surgery, Aristotle University of Thessaloniki, Papageorgiou General Hospital, Thessaloniki, Greece

**Keywords:** Ureterohydronephrosis, paediatric surgery, endoscopic treatment

## Abstract

Obstructive ureterohydronephrosis in childhood population is a matter of debate between paediatric surgeons and paediatricians, as far as the therapeutic protocol that should be applied. Close observation, chemoprophylaxis, endoscopic and surgical approaches are the universally used techniques that provide quality of life in the paediatric patients. Undoubtedly, “the less is more” even when we have to encounter obstructive ureterohydronephrosis in children. Herein, we present a short case series where the endoscopic management of obstructive uropathies proved to be therapeutic without any need of surgical intervention.

## Introduction

Endoscopic management of obstructive uropathies in paediatric population is a challenging field for paediatric surgeons. The surgical approach of ureterohydronephrosis is an established technique which seeks many operating difficulties, especially in children aged under 1-year-old, because of both the disproportion between the size of the dilated ureter and the size of the bladder, the operated bladder exposure to dysfunction and the many other possible postoperative complications [1]. In 2012 and after many controversies in the literature, the British Association of Paediatric Urologists (BAPU) was asked to determine the diameter at which a ureter should be considered as dilated and came into consensus that retrovesical ureteric diameter ≥ 7mm from 30 weeks´ gestation onwards is abnormal [2]. In 1980, King, after Stephens, classified the megaureter into four categories which were then subclassified in primary and secondary reflux megaureter, obstructive megaureter, refluxing megaureter with obstruction and nonrefluxing-nonobstructive megaureter [3]. In 1999, Shenoy and Rance first described the insertion of a temporary Double J (DJ) stent through surgical cystotomy, for the management of two infants who suffered from primary obstructive megaureter (POM) [4]. A year before, in 1998, Angulo *et al*. had described as a definite, less invasive and safe approach the treatment of POM with endoscopic balloon dilatation [5]. Since those first descriptions of endoscopic techniques available, there are few publications in the literature that have reported short series of endoscopically managed paediatric cases with obstructive uropathies of the distal ureter, which seem to result in promising both short-term and long-term outcomes [2]. Here, we present the cases of four paediatric patients who underwent endoscopic treatment for ureterohydronephrosis in our department and their medium-term follow up revealed no recurrence.

## Methods

Below, we present analytically the methods of the four cases of our short case series in order to explain exactly the steps followed. We selected to describe the cases separately in order to eliminate any questions that may arise to the readers.

**Figure 1:**
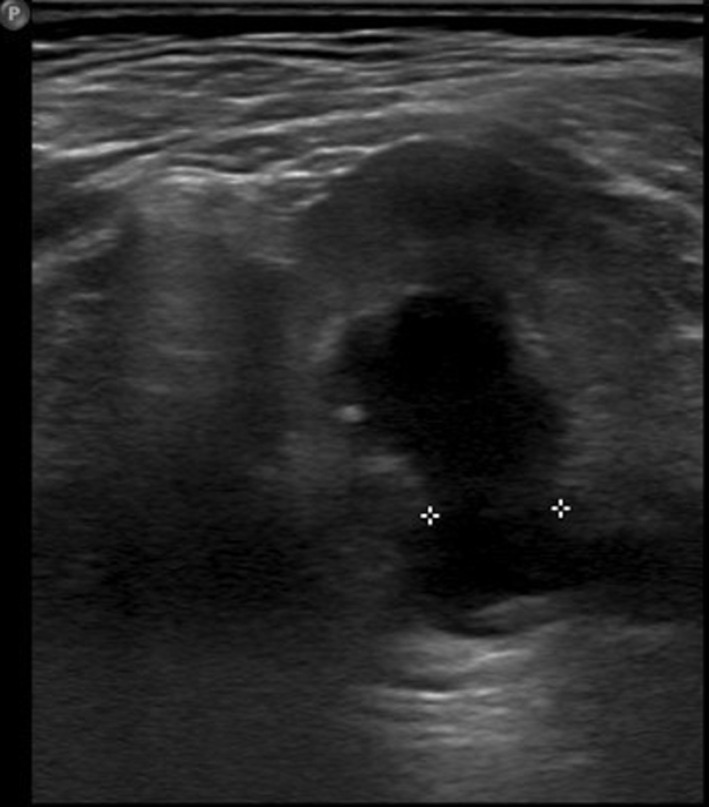
u/s image that shows the dilated ureter just above the vesicoureteral junction

**Figure 2:**
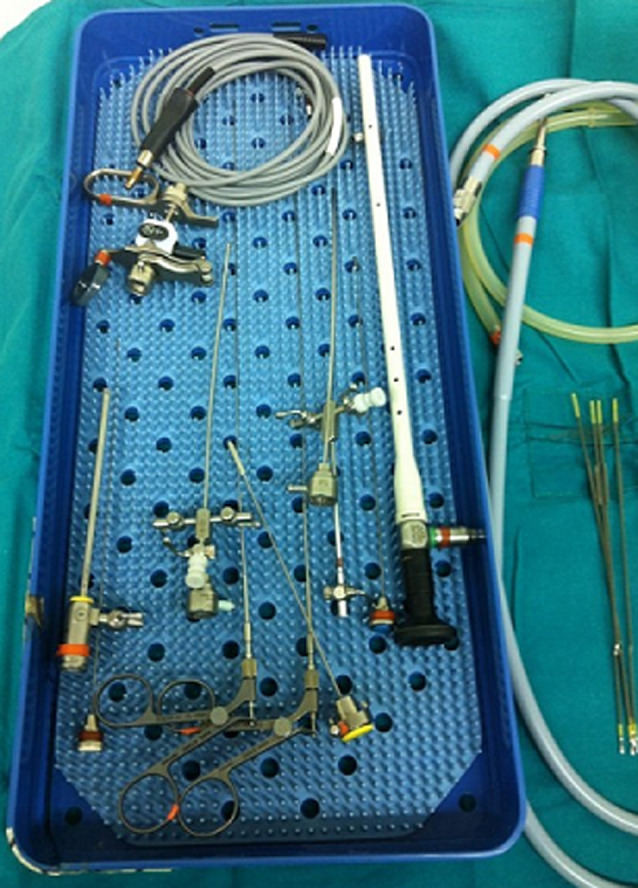
the neonatal cystoscope

**Case #1-child with POM:** in the first case, the patient was an 8-months-old male which was admitted to our outpatient clinic with a diagnosis of ureterohydronephrosis of the left kidney. The u/s was performed after a febrile urinary tract infection in the age of 3-months-old and revealed a dilated renal pelvis (13mm) and a dilated ureter (15mm) (Figure 1). The micturating cystourethrography (MCU) excluded the presence of vesicoureteric reflux (VUR) and the MAG-3 was abnormal (the differential renal function of the left kidney was 34% and the T1/2 max was > 20min). Under general anesthesia, the child underwent cystoscopy with the use of neonatal cystoscope (Figure 2) and we performed endoscopic insertion of a 3Fr DJ catheter in the left ureter with no special maneuvers. It should be underlined that the orifice of the left ureter was in the expected normal positon and during its catheterization we encountered no difficulties.

**Figure 3:**
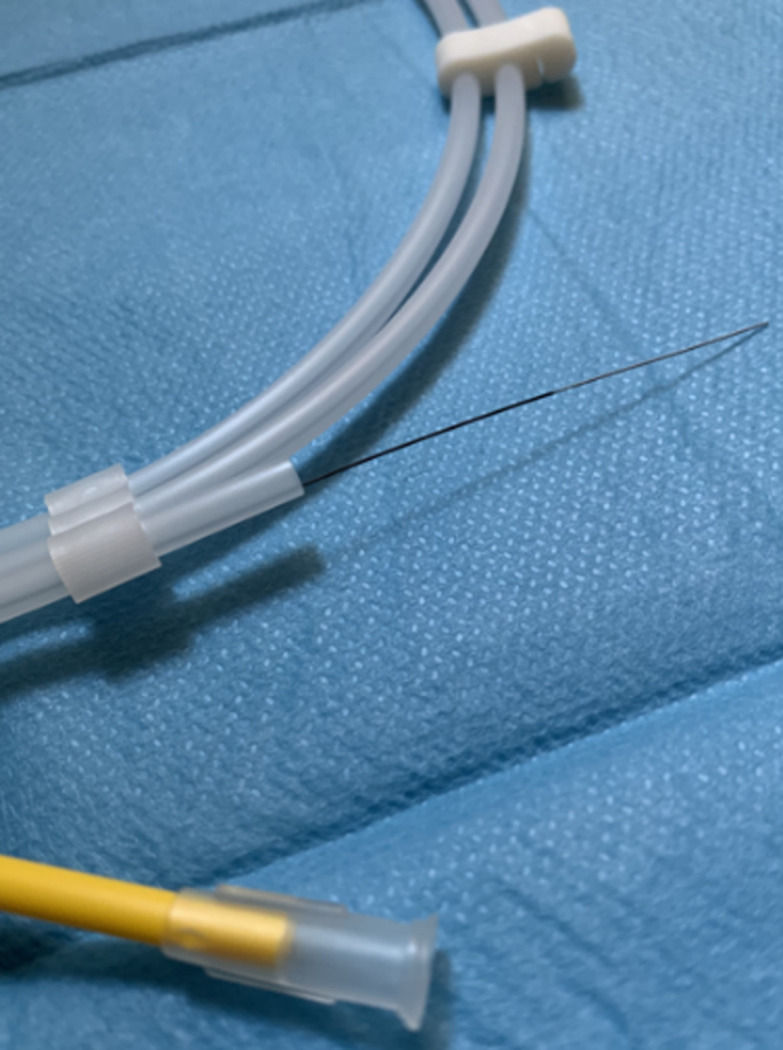
the 0.014-inch diameter hydrophilic guide wire

**Case #2-child with stenosis of the vesicoureteral junction:** in this case, the patient was an 8-months-old male which was admitted to our outpatient clinic with a diagnosis of hydronephrosis of the left kidney. The u/s was performed after a febrile urinary tract infection, which prompt to be pyelonephritis at the age of 5-months-old and revealed hydronephrosis with dilated renal pelvis (16mm) and dilated ureter (18mm). The micturating cystourethrography (MCU) excluded the presence of vesicoureteric reflux (VUR) and the MAG-3 indicated that the differential renal function of the left kidney was 34.8%. Under general anesthesia, the child underwent cystoscopy with the use of neonatal cystoscope. Because of the difficulty in the catheterization of the stenotic ureteric orifice, we, firstly, catheterized it with the use of a hydrophilic guide wire (diameter 0.014 inch) and then, we inserted a 3Fr DJ catheter in the left ureter (Figure 3).

**Case #3-child with stenosis of the vesicoureteral junction:** in the third case, the patient was an 8-months-old male which was admitted to our outpatient clinic with a diagnosis of hydronephrosis of the left kidney. The u/s was performed after a febrile urinary tract infection in the age of 4-months-old and revealed hydronephrosis with dilated renal pelvis (12mm) and dilated ureter (16mm). The micturating cystourethrography (MCU) excluded the presence of vesicoureteric reflux (VUR) and the MAG-3 revealed that the differential renal function of the left kidney was 48%. Under general anesthesia, the child underwent cystoscopy with the use of neonatal cystoscope. Because of the difficulty in the catheterization of the ureteric orifice, we, firstly, catheterized it with the use of a hydrophilic guide wire (diameter 0.014 inch) and then, we inserted a 3Fr DJ catheter in the left ureter.

**Figure 4:**
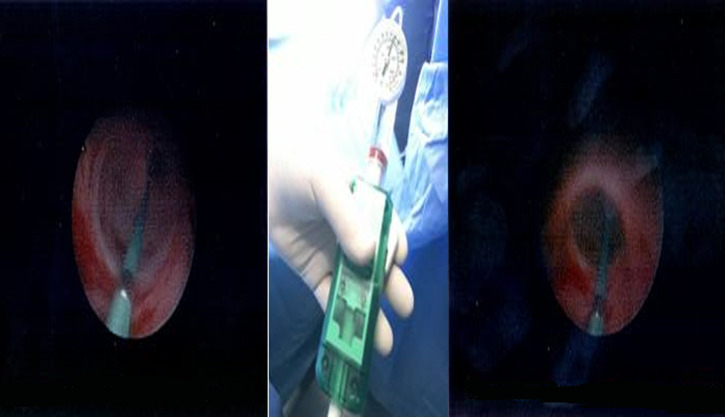
images that show the procedure of the balloon dilation with a 14atm pressure in order to resolve the postsurgical ureteral stenosis

**Case #4-child with post-operative stenosis of the vesicoureteral junction:** in this case, our patient was a 7-years-old female, who had undergone Cohen procedure at the age of three years, because of grade five vesicoureteral reflux. Besides the fact that our patient presented no complications for the next two years after the operation for the VUR correction, the third year had relatively often episodes of abdominal and flank pain without fever. The new kidney u/s revealed hydronephrosis with dilated renal pelvis (34mm) and dilated ureter especially in its proximal and medial part and lesser in its distal part (11mm). The MAG-3 indicated that the differential renal function of the left kidney was 45% and the micturating cystourethrography excluded the vesicoureteric reflux recurrence. We hypothesized the postsurgical strictures presence and we decided to perform endoscopic balloon dilatation of the stenotic lumen of the left ureter. Under general anesthesia, the child underwent cystoscopy with the use of neonatal cystoscope and we first performed endoscopic insertion of hydrophilic wire which diameter was 0.014 inch into the left ureter. It must be underlined that the ureteral orifice was extremely fibrotic and the lumen was extremely difficult to be catheterized. With the use of C-arm, we reassured the wire´s position and continued with the insertion of a 4mm diameter and 15mm length balloon in the left ureter. We expanded the balloon with 14atm pressure for three minutes until the disappearance of the stenotic ring and then we removed it and replaced it with a 10mm diameter and 40mm length balloon which we expanded with 10atm pressure for three more minutes. After those two expansions, we removed the balloon and the hydrophilic wire and we noticed the improvement of the ureteral orifice. We then inserted a 4Fr DJ catheter in the left ureter (Figure 4).

## Results

**Case #1-child with POM:** a day after the endoscopic involvement, the new u/s revealed the decrease in the anteroposterior diameter of the renal pelvis (8.2mm) and a less significant decrease in the ureter´s diameter (13mm). The next day the child was discharged in excellent general condition and guides for chemoprophylaxis uptake. The third day after the endoscopic treatment, the child presented febrile urinary tract infection, reassured by positive urine culture which revealed pseudomonas aeruginosa and was treated successfully with antibiotics. A month and three months postoperatively, the new u/s revealed that the diameter of the left renal pelvis was 5mm and the diameter of the ureter just above the vesicoureteral junction was in normal values. Six months after the endoscopic insertion of the DJ, a DMSA revealed normal findings (the differential renal function of the left kidney was 50%). We have to mention that the preoperative MAG-3 was performed quite closely to a urinary tract infection episode and this is the possible explanation of the postoperative increase in the differential renal function of the left kidney. At the seventh month after the operation and after a u/s of the urinary tract which did not reveal abnormal findings, we removed the DJ. The child during the next two years had no pathological findings from the u/s and radioisotopic investigations and did not present any episode of febrile urinary tract infection.

**Case #2-child with stenosis of the vesicoureteral junction:** a day after the endoscopic involvement, the new u/s revealed the decrease in the anteroposterior diameter of the renal pelvis (8.2mm) and a less significant decrease in the ureter´s diameter (13mm). The next day the child was discharged in excellent general condition and guides for chemoprophylaxis uptake. A month and three months postoperatively, the new u/s revealed that the diameter of the left renal pelvis was 6mm and then 4mm respectively and the diameter of the ureter just above the vesicoureteral junction was in normal values (5.8mm and 5mm). Six months after the endoscopic insertion of the DJ, the child presented with fever and was hospitalized in our department. The new u/s revealed a dilated left renal pelvis (10.6mm) and dilated ureter (14mm). Besides the negative urine culture, we decided to remove the DJ under general anesthesia and indeed, the DJ culture was positive. The child was treated with IV antibiotics for a week and was discharged after 9 days of hospitalization in a good general condition with guides for chemoprophylaxis use. Ten months after the first operation, a new MAG-3 revealed deterioration of the differential renal function of the left kidney, which was 32%. The decrease in the rate could be attributed to the previous described postoperative febrile urinary tract infection. We decided not to operate endoscopically, but to use the “wait and watch” technique while the child was under chemoprophylaxis. The little patient during the next two years had no episodes of urinary tract infection and presented no deterioration from the u/s and radioisotopic investigations.

**Case #3-child with stenosis of the vesicoureteral junction:**a day after the endoscopic involvement, the new u/s revealed the decrease in the anteroposterior diameter of the renal pelvis (7mm) and a significant decrease in the ureter´s diameter (6mm). The next day the child was discharged in excellent general condition and guides for chemoprophylaxis uptake. A month and three months postoperatively, the new u/s revealed that the diameter of the left renal pelvis was 5mm and the diameter of the ureter just above the vesicoureteral junction was in normal values. Six months after the endoscopic insertion of the DJ, a DMSA revealed normal findings (the differential renal function of the left kidney was 50%). At the seventh month after the operation and after a u/s of the urinary tract which did not reveal abnormal findings, we removed the DJ. The child during the next two years had no pathological findings from the u/s and radioisotopic investigations and did not present any episode of febrile urinary tract infection.

**Case #4-child with post-operative stenosis of the vesicoureteral junction:** after the endoscopic balloon dilatation of the stenotic lumen, the patient was discharged 5 days postoperatively, in excellent general condition. A month after the operation and after a normal kidney u/s with no dilation marks, we removed the DJ endoscopically. Three, six, nine, twelve and eighteen months after the operation, the u/s findings were in normal values.

## Discussion

In our paediatric surgery department, we managed the four previously described cases of obstructive uropathies causing ureterohydronephrosis which etiology was the most frequent encountered in paediatric patients. In our knowledge and besides the many categorizations in the literature, the stenosis of the vesicoureteral junction, the primary obstructive megaureter and the post-operative stenosis of the vesicoureteral junction due to strictures formation represent the three main causes of ureterohydronephrosis in children. Herein, our team reports and describes the acquired knowledge that resulted from endoscopic treatment of obstructive uropathies in our department and can contribute to the differentiation between organic and functional obstruction cystoscopically. To our knowledge, while the catheterization of the pathologic orifice is always easy and demands no special maneuvers in the cases of POM, the same procedure in the vesicoureteral junction stenosis is mainly difficult, demands special maneuvers and almost in all cases the insertion of a 0.014 inch diameter hydrophilic guide wire before the insertion of the DJ. We propose the cystoscopy and orifice catheterization as a both diagnostic and therapeutic procedure in obstructive uropathies in cases of organic and functional obstruction. Endoscopic management of paediatric obstructive uropathies that cause megaureter is an upcoming field, since the first description of Shenoy and Rance in 1999 [4]. This promising technique gives the opportunity to the paediatric surgeon to postpone or even avoid an open surgical intervention especially in children under 1-year-old, at whom the open bladder operation for ureteral reimplantation, with or without ureteral remodeling, is both challenging and potentially harmful because of the disproportion of the bladder-ureteral size [1, 2].

The BAPU agreed in 2014 that ureteric reimplantation below 1 year of age is feasible, but the majority of the group proposed the consideration of alternative temporizing or permanent interventions (temporary DJ stenting, endoscopic balloon dilatation, endoureterotomy and cutaneous ureterostomy) and avoidance of reimplantation in infancy [2]. According to BAPU, indications for surgical intervention include an initial differential renal function < 40% especially when it is associated with excessive hydroureteronephrosis and failure of conservative management (febrile urinary tract infections, pain, worsening dilatation or deteriorating differential renal function on serial scintigraphy scans) [2]. In our cases none of the children demanded post endoscopically surgical repair. Teklali Y *et al*. proposed the inclusion criteria for endoscopic treatment in 2018 as the presence of symptoms and significant ureteral dilatation > 10mm or marked renal dysfunction on scintigraphy [6]. It is underlined as a matter of debate in the literature, that the endoscopic intervention may be a treatment choice in asymptomatic patients that present impaired renal function on the renal scintigraphy [7]. Once the endoscopic treatment is chosen, the DJ stent remains in the ureter for approximately 2 months while prophylactic antibiotics are administered [6]. We should refer that besides this recommendation, we decided to keep the DJ for a longer, urinary tract infection free and without major complications period in our patients, fact that can be considered as overtreatment. Our decision is supported by the knowledge that in 50% of the cases, spontaneous and complete resolution of megaureter occurs in children before the age of three [6]. After the endoscopic removal of the DJ, the follow up indicates that the child must undergo ultrasound of the urinary collecting system 3, 6 and 12 months after the endoscopic intervention and then once a year. As far as the renal scintigraphy, MAG-3 should be performed 6 months post operatively [8].

Our team is in compliance with Y Teklali *et al*.criteria for stopping follow up, which are an asymptomatic patient two years or more after treatment and a retrovesical ureteral diameter <6mm [6]. We furthermore propose that the child should be closely checked by a paediatric nephrologist and to undergo a u/s annually until the age of 18 in order to secure a normal kidney function. Besides the fact that minimal invasive endoscopic treatment is fairly proved as safer for young children as a first step approach, it is not complication-free according to the literature [6]. Ureteral and bladder injury, poor tolerance of the DJ stent which presents as flank pain, DJ migration which demands repositioning, upper and lower urinary tract infection, vesicoureteral reflux and persistent hematuria are the main complications encountered in the bibliography [2, 6, 9]. In our short series, two of the four children presented febrile urinary tract infection which in the case #1 resolved with IV antibiotics and in case#2 demanded the removal of the DJ catheter. As far as the endoscopic balloon dilatation technique use for postsurgical strictures of the urinary tract, poor data exist in the literature [10]. Although, the procedure is extremely challenging and expensive, the promising results may predict the techniques´ establishment during the next decade [10]. We underline the advantages of this technique in our case#4, because it was proved to be painless and complication-free in our little patient who tolerated it well and did not have to undergo a technically difficult and painful second operation after a previous major injury surgery (Cohen procedure). The main complication after the endoscopic balloon dilatation technique is the (re)appearance of vesicoureteral reflux which can be diagnosed with the use of MCU [11]. We did not choose to use MCU postoperatively, because our patient remained asymptomatic and we did not consider as appropriate to perform an invasive diagnostic examination which could trigger a non-willing urinary tract infection.

## Conclusion

Endoscopic treatment of obstructive hydroureteronephrosis in children especially under 1 year is a safe and effective method. Undoubtedly, there are difficulties in the catheterization of the ureteral orifice but the appropriate equipment and the surgeon´s experience are the key for a positive and satisfactory outcome. We state that the number of our patients and the duration of follow-up is not safe for drawing final conclusions.

### What is known about this topic

- Surgical approach of ureterohydrone phrosis is a technique which seeks many operating difficulties, especially in children aged under one year;
- Minimal invasive endoscopic treatment is fairly proved as safer for young children as a first step approach but it is not complication-free.

### What this study adds

- Our study shows that the catheterization of the pathologic orifice is always easy and demands no special maneuvers in the cases of POM;
- Our study shows that the catheterization of the stenotic orifice in the vesicoureteral junction stenosis is mainly difficult, demands special maneuvers and almost in all cases the insertion of a 0.014 inch diameter hydrophilic guide wire before the insertion of the DJ;
- We propose the cystoscopy and orifice catheterization as a both diagnostic and therapeutic procedure in obstructive uropathies in cases of organic and functional obstruction.
